# Early Word Recognition and Later Language Skills

**DOI:** 10.3390/brainsci4040532

**Published:** 2014-10-24

**Authors:** Caroline Junge, Anne Cutler

**Affiliations:** 1 Utrecht University, Heidelberglaan 1, 3584 CS Utrecht, The Netherlands; 2 University of Amsterdam, Weesperplein 4, 1018 XN Amsterdam, The Netherlands; 3 MARCS Institute, University of Western Sydney, Locked Bag 1797, Penrith, NSW 2751, Australia; E-Mail: A.Cutler@uws.edu.au; 4 Max Planck Institute for Psycholinguistics, Wundtlaan 1, 6525 XD Nijmegen, The Netherlands; 5 Donders Centre for Brain, Cognition and Behaviour, Radboud University Nijmegen, Geert Grooteplein-Noord 21, 6525 EZ Nijmegen, The Netherlands

**Keywords:** speech segmentation, word recognition, individual differences, longitudinal

## Abstract

Recent behavioral and electrophysiological evidence has highlighted the long-term importance for language skills of an early ability to recognize words in continuous speech. We here present further tests of this long-term link in the form of follow-up studies conducted with two (separate) groups of infants who had earlier participated in speech segmentation tasks. Each study extends prior follow-up tests: Study 1 by using a novel follow-up measure that taps into online processing, Study 2 by assessing language performance relationships over a longer time span than previously tested. Results of Study 1 show that brain correlates of speech segmentation ability at 10 months are positively related to 16-month-olds’ target fixations in a looking-while-listening task. Results of Study 2 show that infant speech segmentation ability no longer directly predicts language profiles at the age of five. However, a meta-analysis across our results and those of similar studies (Study 3) reveals that age at follow-up does not moderate effect size. Together, the results suggest that infants’ ability to recognize words in speech certainly benefits early vocabulary development; further observed relationships of later language skills to early word recognition may be consequent upon this vocabulary size effect.

## 1. Introduction

Most of the words that infants hear in their first year are embedded in multi-word utterances, without clear pauses to signal word onsets [1,2,3]. Even in semi-natural play sessions in which parents are instructed to teach their infants certain words, the target words rarely occur in isolation [4,5]. Thus, it is clear that finding the words making up a speech stream, and remembering them too, are prerequisites for lexicon construction.

Indeed, recent studies reveal a linear relationship between infants’ ability to recognize words from continuous speech in a laboratory setting, and the subsequent size of their lexicons [6,7,8,9]. The present paper further assesses the importance of infants’ ability to segment words from speech by investigating, in new ways, the language profiles of two sets of children who as infants participated in segmentation studies, and who had not previously been post-tested [10,11]. These findings will then be contrasted with earlier studies by incorporating them into a meta-analysis on the relationship between infant speech segmentation studies and the children’s subsequent language development.

Recognizing words from continuous speech is a non-trivial task, since even in child-directed speech, there are no reliable pauses in the speech signal that correspond to word onsets. Earlier behavioral research has revealed a variety of cues in the native language that infants can use to detect words in a speech stream, although all such cues are probabilistic rather than deterministic: no single cue is sufficient to detect word boundaries [12]. These cues follow different developmental trajectories, with six months as the earliest behavioral evidence that infants can find words. Most evidence about the developmental trajectories of speech cues to find words in speech stems from behavioral findings that used the head turn preference (HTP) technique. (Note, however, that electrophysiological evidence suggests that infants can find words at an earlier age than commonly follows from the HTP findings [7,13]; nevertheless, although future ERP studies might reveal that language-specific trajectories begin earlier, the relative onset and weighing of each segmentation cue should follow similar patterns as is observed in the HTP experiments.) At six months, American-English infants can use a highly frequent name such as “mommy” or their own name as an anchor to start detecting subsequent words [14], but they cannot yet otherwise start recognizing monosyllabic words [15]. Infants’ sensitivity to a prosodic cue such as the dominant stress pattern develops around 7.5 months [16], while their sensitivity to phonotactic cues has been demonstrated to be present around nine months [17].

Across languages, differences have been revealed in whether and when infants show sensitivity to a particular cue (for a recent review see [18]). Take for instance the case of word stress, which is a reliable cue for infants from stress-based languages: a stressed syllable signals word onset for the majority of polysyllabic words [19]. In contrast, for infants acquiring syllable-based languages, a stress cue should not be reliable, and indeed, Parisian-French infants appear less sensitive to this cue [20]; though see also [21]. However, even for infants from similar language backgrounds, there are cross-linguistic differences: German and Dutch infants only succeed in using word stress as a possible segmentation cue from nine months of age ([22,23], respectively), whereas American-English infants achieve this by 7.5 months. Houston *et al*. [23] interpret this asymmetry in terms of the relative salience of the difference between stressed and unstressed syllables (greater in American-English than in Dutch or German). All in all, the studies discussed above indicate that infants gradually become aware of the myriad of cues that are available in their ambient language, with some cues being easier to notice than others, dependent on their native language. Hence, these differences reflect that each cue must have been acquired, through increased experience with the native language. Increased listening experience also helps infants to suppress information irrelevant for word recognition: 7.5-month-olds fail to recognize familiarized words now uttered by a different speaker [24] or with different speech affect [25] or pitch [26], and all listeners need to eventually learn that such changes should not disrupt the word identification process. The general developmental trajectories of speech segmentation cues thus became apparent by comparing studies that have tested various groups of infants that differed in age or in language backgrounds. However, even within group averages there is individual variation that reflects the infant’s aptitude to find words in the speech stream. Given that these studies reflect the infant’s ability to use a particular cue learned from the ambient environment, this learnability is both shaped by the amount and type of input the infant receives, and by their ability to detect (language-specific) patterns. Evidence for the role of input comes from examining semi-spontaneous infant-directed speech samples: infants who receive relatively more multi-word utterances develop larger vocabularies, presumably mediated through better speech segmentation skills [5]. Type of input also immediately affects word learning: after listening passively to an artificial speech stream, 17-month-olds were only able to learn novel words when these mapped on to whole words from that speech stream, but not when they mapped to part-words [27]. This further underscores how vital it is to first recognize words in running speech for making a successful word-object mapping.

There is also growing evidence that infants’ laboratory performance in speech segmentation tasks is related to vocabulary development. In a seminal study, Newman and colleagues were the first to show this link [8]. They examined subgroups of two-year-olds with extreme vocabulary sizes (the top and bottom 15% of a large cohort) on a variety of infant speech perception tasks, measuring language discrimination, prosodic preferences and speech segmentation, and found that the two sub-groups differed mainly in their segmentation ability as infants, with high-vocabulary toddlers having shown, as infants, significantly stronger evidence of recognizing words from continuous speech. At the time of a later assessment at five years, the relationship with early segmentation was still apparent: children who as infants had shown evidence of segmentation had higher current language skills, and higher language-skill ratings by parents, than those who had shown no evidence of segmentation ability.

This important finding has now repeatedly been replicated, both with American-English infants [9] and Dutch infants [6,7], and with electrophysiological [6,7] as well as the behavioral [8,9] indices of speech segmentation ability. Table 1 summarizes all reports to date.

Behaviorally, speech segmentation ability is typically measured in a head turn preference (HTP) paradigm, with a familiarization phase followed by a test phase [16]. The test phase compares infants’ listening times for familiarized words versus control words. Words are presented in multi-word utterances (during familiarization or at test, or even both), so that the appearance of differential responses at test for familiar versus unfamiliar words entails that infants have recognized the familiarized words, and thus must have segmented them from the continuous speech signal. With natural speech samples, infants generally prefer familiarized words at test over control words (and the more pronounced this preference, the larger are vocabularies at two years [9]).

**Table 1:** Overview of studies that reports infant speech segmentation ability to predict future language scores.

Author & Year	Design	Infant Age	Segmentation Measure(s)	Follow-Up Age(s)	*N*	Follow-Up Measure	Expected Direction	Effect Size
Newman *et al*. (2006) [8]	Group contrast	7.5–12	HTP (variety of tasks)	a. 24	77	CDI	+	+0.318 *
				b. 55.5	27	TOLD-P3	+	+0.439 **
Junge *et al*. (2012) [6]	Correlation	10	ERP	a. 12	28	CDI	−	−0.564
				b. 24	28	CDI	−	−0.383
Singh *et al*. (2012) [9]	Correlation	7.5	a. HTP (simple task)	40	24	CDI	+	+0.32
			b. HTP (complex task)				+	+0.51
Kooijman *et al*. (2013) [7]	Correlation	7	ERP	36	23	Reynell	−	−0.474

Note: Design indicates whether the relationship is observed in a group contrast or in a correlation. Ages are reported in months. Segmentation measure relates to whether segmentation ability was measured behaviorally (HTP; head-turn preference) or electrophysiologically (ERP; event-related potentials). *N* = sample size of infants seen at both initial testing and follow-up. Expected direction reflects whether relationship was expected to be positive or negative. Effect sizes are reported as untransformed *r*; * indicates that it was calculated from the exact *t*-value (*cf*. [28] p. 71), and ** indicates that it reflects the φ-coefficient (*cf*. [28] p. 74) that we obtained from the reports on the categorical data.

Electrophysiological markers of infant speech segmentation skill are obtained by comparing event-related potentials (ERPs) time-locked to familiarized and control words in continuous speech. The first ERP demonstration of a segmentation response was by Kooijman and colleagues with 10-month-olds acquiring Dutch [10]. The recognition response was a word familiarity effect (WFE) with a negative polarity, predominantly present on left frontal electrodes. Such a response appears quite stable for this age group: similar effects appeared in other 10-month-old word-segmentation studies in our laboratory [6,11,29], and in French [30], and German 12-month-olds [31]. Crucially, the WFE also appears stable at the individual level: when both familiarization and test involve continuous speech, infants who show a WFE at test also show this effect in the familiarization phase, within six word repetitions across different utterances [11].

The WFE’s links to later language development have been demonstrated in several ways. First, the larger the WFE in 10-month-olds who heard a word once in an utterance, the more words the same infants understood at 12 and 24 months [6]. Second, although younger infants generally show a familiarity effect with positive polarity (Dutch seven-month-olds: [7]; German six-month-olds: [31]), those seven-month-olds with a more mature, negative-going, effect (‘Negative Responders’) displayed higher language scores at three years than their peers with a positive familiarity effect (“Positive Responders”; [7]). Studies on infant discrimination of phonetic contrasts or recognition of known versus unknown words also report meaningful variation in the polarity of infant ERP responses, with a negativity generally linked to more mature processing (phonemes: [32,33]; known words: [34,35]). Here, too, we see that a negative response, in this case for phonetic discrimination, in the same age group is linked to better vocabulary development [36]. The difference in polarity has been suggested to reflect different listening modes; with a positivity indicating acoustic processing, and a negativity reflecting a more advanced, linguistic processing of the speech stream (*cf*. [7,36]). Together, these studies further point to the predicative value that infant ERPs can have in reflecting a child’s relative language experience or language maturity. For speech segmentation abilities, it is clear that the more negative the WFE, the more advanced the child’s current or future language development [6,7,11].

Thus both behavioral and electrophysiological markers of infants’ ability to detect word boundaries in speech have been linked to subsequent language development. By definition, longitudinal studies are time-consuming. As noted by a recent meta-analysis on infant speech perception skills [37], however, follow-up measures in consequence have nearly exclusively been parental checklists (*i.e.*, MacArthur-Bates Communicative Development Inventory; CDI: [38]; for Dutch, N-CDI: [39]) that were returned when infants turned two. This has made for far less variation in outcome measures and ages than among the predictor measures. To put the relationship between early speech segmentation ability and future language development therefore to a more direct test, we here examine links between results from two infant segmentation studies ([10,11], respectively), and the participants’ later language development, using different follow-up procedures at different ages from those used in prior literature.

In Study 1, our follow-up measure is a processing task. We re-tested the 10-month-olds from [11]: this study had shown that when multi-word utterances were used both in familiarization and at test, the WFE proved consistent across phases within subjects (*i.e.*, the infants who showed a large negative effect at test had also shown such an effect during familiarization). Now, all infants bar one returned to our lab at the age of 16 months; by this time their mastery of an initial vocabulary can be assumed. This is younger than the age for which outcome measures are commonly reported, so we expect to find clear correlations. Any language task that is a valid measure of current language development should show such a relationship, and we chose the behavioral measure of word recognition in real time known as looking-while-listening (LWL, *cf*. [40]). This procedure is based on the finding that infants look longer at a visual stimulus that matches audio they hear than at one that does not [41,42]. Infants’ looking behavior can be taken as an index of current language development, because the longer infants look at the correct picture upon naming, the better their vocabulary skills (e.g., [43]). Using the LWL-task, Fernald and colleagues [44,45,46] demonstrated that individual differences in infants’ speech processing efficiency for known words at 18–25 months are related to their later level of lexical and grammatical development up to eight years. Behavioral indices of known word recognition have also been linked to present vocabulary size (e.g., [43,47,48]; but see [49,50]. In our version of the LWL-task, infants saw pairs of known objects (e.g., a cow and a dog) and pairs of novel objects. Here we present only data on the known word condition. (While an advantage of having a six-month period for follow-up testing is that it increases the likelihood that infants return for testing—e.g., have not yet moved out of the area—little time was available to pilot word learning studies. We modeled our novel word learning study on a study by Swingley and Aslin [51], in which 18-month-olds speaking the same language robustly learned novel words. Pilot studies however revealed that 16-month-olds do not yet learn novel words in the way observed for 18-month-olds [51]. We tried simplifying the paradigm by adding a play phase with the novel items, but the large majority of the infants still did not show evidence of novel-word learning. We therefore focus only on links between eye tracking responses to known words and ERP segmentation responses.) Target words were presented in continuous speech, so that as in the task performed at 10 months, segmentation was once more a necessary step for successful word recognition.

Research so far suggests that infant markers of speech segmentation abilities predict future language skills up to age three for an entire distribution of infant participants [6,7,9], and up to age five for the extremes of such a distribution (*i.e.*, top and bottom 15%; [8]). Although the latter approach maximizes the chances of finding group differences, Newman and colleagues [8] acknowledge that it does not provide a full picture of the relationship, in that it disregards the 70% of children falling neither in the top or bottom ranks [9]. It remains therefore unclear whether infant markers of speech segmentation continue to directly predict language proficiency observed in older children and adults. Although ideally we would have followed the same children at multiple time points, to track links with earlier speech segmentation over time, this was not feasible. In Study 2, we therefore retested another sample of participants from a study showing a WFE at 10 months, indeed from the study in which this effect was first observed [10]. At return, the original 10-month-olds were now around five years old. To assess their current language profiles, we used the same standardized language tasks and parental questionnaires as in the seven-month/three-year comparison [7].

In both studies, infants had participated in an ERP task at 10 months, with multiple familiarization-and-test blocks. In Study 1, both familiarization (eight different sentences around the same word) and test (four sentences; two containing the familiarized word, two containing a matched control word) involved continuous speech, whereas in Study 2, a test phase of continuous speech was preceded by a familiarization phase of 10 isolated word tokens. Across familiarization phases, ERPs to word repetitions became gradually more negative. Similarly, in both test phases, the 10-month-olds on average displayed a WFE with negative polarity (familiarized versus control asymmetry) around 400 ms from word onset. Comparison of individual performance in the 10-month-old groups revealed that although most had shown the standard WFE (negative polarity), some participants had shown an effect with positive polarity, *i.e.*, resembled a younger age group. To examine the links with future language profiles, we therefore created for each study again two subgroups based on the polarity of their recognition effect at test (advanced recognizers are the Negative Responders; less-mature infants are the Positive Responders).

Recall that for Study 1 we have already shown that the WFE is related to current language processing; that is, we compared this recognition effect from the test phase with recognition effects over the course of the familiarization phase (contrasting the first pair of targets with subsequent pairs): the Negative Responders initiated a similar mature WFE within five to six presentations, whereas the Positive Responders did not show any effect of recognizing target words within eight presentations [11]. Hence, for this group of infants, their WFE at test had concurrent predictive value on the number of tokens required to elicit a recognition response in the familiarization phase. To further bolster our claim that infant speech segmentation skill is a sensitive measure for concurrent linguistic processing ability, Study 2 therefore first compares subgroups on their building of a memory trace in the familiarization phase. We then continued to assess the relationship between infant WFE and markers of future language processing. If segmentation ability in infancy predicts older children’s language skill, we expect Negative Responders to exhibit higher language scores than Positive Responders. Finally, in Study 3 we conduct a meta-analysis, incorporating both the current results and other results from the literature, to examine the extent to which the present results are in line with earlier research pointing to a link between infants’ ability to find words in running speech and their later success in constructing a lexicon.

## 2. Study 1: The Infant WFE and LWL at 16 Months

In the original study [11], 10-month-olds participated in an ERP study assessing their ability to find words within multi-word utterances, with continuous speech presented both in the familiarization (eight unique utterances) and test phase (four unique utterances, two containing the familiarized word and another two containing a control word). Results from the test phase showed that a majority of infants (“Negative Responders”; *n* = 19; 11 girls) showed a mature WFE with negative polarity at left frontal electrodes (F7, F3, C3, FC1, FC5) for target words relative to control words for a prolonged time (220–900 ms from target onset). Over the course of the familiarization phase, these Negative Responders (defined on the basis of the test phase results) displayed a similar negative recognition response within the first six repetition sentences, whereas Positive Responders (*n* = 9; five girls) failed to show any recognition response at this stage of familiarization. Thus it appears that the WFE proves constant at the individual level. Using the original classification of Negative *vs*. Positive Responders, we now follow the same infants, and measure whether the groups continue to differ in their on-line efficiency of language processing, this time at 16 months.

### 2.1. Methods

#### 2.1.1. Participants

In the original study [11], there were 28 monolingual Dutch-acquiring 10-month-olds (mean age = 307 days, range 293–319 days; 16 girls), coming from families with no history of language problems. Of this sample, 27 children returned at 16 months. One girl did not remain seated on her parent’s lap, so we have eye tracking data of 26 children (92.9%; 15 girls). At return, they were on average 16; 01 months (range 480–512 days). According to the classification of their infant ERP responses at test (*cf*. [11]), there were 8 Positive Responders (5 girls) and 18 Negative Responders (10 girls; the single drop-out would also have been included into the latter subgroup); the two subgroups did not differ in age at 10 or at 16 months (*t*_24_ < 1). Parents signed informed-consent forms, and filled in N-CDIs [39], and children received a book after the experiment.

#### 2.1.2. Stimuli

We selected six referents as presumably known: four animate referents (baby, cat, cow, dog) and two inanimate objects (car, ball). Parents were asked to estimate how well their child would understand each known word on a scale from 1 (definitely not) to 5 (definitely yes). Mean ratings for each word ranged between 4.12 (for cow) and 4.72 (for ball), indicating that these were indeed well-known words to the infants. The visual stimuli, presented on the screen in the eye-tracking task, consisted of digitized photographs against a dark grey background. In the test phase, each object was always yoked with the same object: cow with dog, cat with baby, and car with ball. Each pair was from the same general semantic category, and words in a pair did not share any phonological overlap. To reduce repetition of pictures, we used three different picture tokens for each known referent.

The auditory stimuli were digitally recorded in a soundproof booth, sampled at 44.1 kHz mono to disk. A female native speaker of Dutch (different from the one in the ERP experiment) uttered the stimuli in a child-directed manner. For each of the known trials in the familiarization phase, infants heard a token of the carrier phrase “Kijk een…” (“Look, a …”), followed by the target (*i.e.*, [kʊ] “cow”; [hɔnt] “dog”; [pʊs] “cat”; [bebi] “baby”; [ɔuto] “car”; and [bɑl] “ball”. To avoid too much repetition for the sentences in the test phase, we used three different carrier sentences per target: “Zie je de [target]?”; “Waar is de [target]?”, “Kijk naar de [target]!” (“Do you see the [target]?”; “Where is the [target]?”; “Look at the [target]!”). Note that the target words were thus first heard in continuous speech, although offsets were aligned with an utterance edge [52]; infants had to segment them from the preceding context. Mean target word duration within test sentences was 736 ms (SD = 117, range 578–896). Target words were also recorded in isolation, with mean durations of 660 ms (SD = 147, range 382–789).

#### 2.1.3. Procedure

Infants were tested in a dimly lit experimental room with a Tobii 1750 eye tracker (Tobii 1750; Tobii Technology, Stockholm, Sweden), with a 50Hz sample rate and a 9-point calibration procedure. Each infant sat on a parent’s lap, with the eye tracker positioned about 60 cm in front of the infant’s face. The parent wore head-phones and heard masking music, and was instructed not to distract the child by interacting in any way.

The experiment started when calibration for at least eight out of nine points was successful. The eye-tracking task consisted of two blocks: a familiarization phase followed by a test phase. During familiarization infants saw each known object in isolation once, for three seconds. After 500 ms they heard “Look, a [target]”. The test phase comprised 18 known trials. Each trial began with the simultaneous presentation of two pictures, positioned at the left and the right of the screen and centred vertically. The two pictures remained on the screen until the end of the trial at 5000 ms. The auditory stimulus was played such that the first onset of the target word started at 2500 ms, and that the second token (in isolation) followed 750 ms after the sentence; for instance, “Do you see the dog? Dog!”.

Because of our focus on individual differences, we used a consistent order of trials to avoid variation that might arise from different novel pairings or item orders. We counter-balanced within subjects the target position (left, right) and carrier sentence. Each picture token served equally often as a target and a distractor. Trials occurred pseudo-randomly, with the restriction that the same picture was never presented twice in a row. We also played attention grabbers (e.g., an expanding circle accompanied by cheery music) after every three to four trials. The experiment lasted about six minutes. After the experiment, parents indicated which words their child produced from the infant version of the N-CDI ([39]: 434 words divided over 19 semantic categories; mean = 24 words; range 0–116; not normally distributed: skewness: +2.18; kurtosis: +6.38).

#### 2.1.4. Data analysis

Trials were included only when infants fixated both objects prior to hearing a matching label, that is, where we could be sure that subsequent looking behavior was driven by acoustic word recognition, and not by an object’s novelty. We then labelled trials based on which object the infants were consistently fixating at word onset (in the 200 ms time window from target word onset): either as target-initial (mean = 4.0, range 2–11), or as distractor-initial (mean = 5.5 trials, range 2–9). For each trial we obtained two measures: the duration of first look (FL) as well as the proportion of looking time at target after naming (PTL; from 360 to 2500 ms after naming, *cf*. [49]). LWL studies typically report both accuracy and reaction time as measures of speech processing efficiency. Although the latter measure is clearly defined as the mean reaction time to shift from the distracter to the target (*i.e.*, from first look durations in distractor-initial trials; [40]), accuracy can be calculated in a variety of ways. For instance, Fernald and colleagues use the relative proportion of target looking (*i.e.*, PTL) for both target- and distractor-trials (e.g., [44,46]), but accuracy has also been based on absolute fixations such as on durations of first or longest look [53,54]. On target-initial trials, the correct response would be to continue fixating the target, but on distractor-initial trials it would be to shift gaze to the target [40,45,54]. Besides the PTL measure, we here also use the looking time difference in first looks for target- versus distractor-initial trials as a measure of accuracy: a positive difference reflects that upon naming, infants overall fixate targets longer than distractors. Hence, for target-initial trials, the latency of first looks (FLtarget) reflects infants’ ability to sustain attention, with larger looks reflecting longer fixations at target, whereas for distractor-initial trials, first looks (FLdistractor) reflect their reaction time to switch to correct target. Positive and Negative Responders are compared on these three measures.

### 2.2. Results

Overall, infants understood the labels for known words. Upon hearing a name, their relative total target looking times was above chance (*i.e.*, compared to 0.50; PTL = 0.546, SD 0.09; *t*_25_ = 2.63; *p* = 0.014; 95% CIs [0.51; 0.58]). Similarly, their first looks were longer for target-initial trials than for distractor-initial trials: the mean looking time difference is +123 ms (SD 310 ms), which is above zero (*t*_25_ = 2.02; *p* = 0.027; 95% CIs [−3; +248], one-tailed; *cf*. [54]). When infants fixated the target at naming, they continued to look for another 1035 ms (SD 273), whereas when they fixated the distractor, they switched gaze faster, at 913 ms after target onset (SD 237).

The two subgroups with respectively a mature negative *vs*. less-mature positive WFE in the segmentation task at 10 months differ in both of their processing measures reflecting accuracy. Negative Responders fixated target on average 0.579 (SD 0.073), which is significantly above chance (*t*_17_ = 4.62; *p* < 0.001, 95% CIs [0.54; 0.61]). Positive Responders on the other hand performed at chance (mean 0.472, SD 0.082; *t*_7_ = −0.96; *p* = 0.37, 95% CIs [0.40; 0.54]). This difference between Negative and Positive Responders in proportional target looking time after naming is significant (*t*_24_ = 3.33; *p* = 0.003; 95% CIs [0.04; 0.17]).

Similarly, the groups differed on their looking time difference for first looks, as measured by a paired *t*-test (*t*_24_ = 2.22; *p* = 0.036; 95% CIs [+19; +525]). Whereas Negative Responders showed evidence of understanding words (mean looking time difference +207 ms; *t*_17_ = 2.87, *p* = 0.011; 95% CIs [+54; +359]), Positive Responders showed no such effect (mean looking time difference −67 ms; *t*_7_ = −0.78; *p* = 0.46; 95% CIs [−266; +135]). Subsequent paired *t*-tests reveal that it is first look duration for target-initial trials (*i.e.*, FLtarget; *t*_24_ = 3.42; *p* = 0.002; 95% CIs [+132; +533]), but not that for distractor-initial trials (FLdistractor; *t*_24_ = 0.59; *p* = 0.56; 95% CIs [−151; +270]) that distinguishes the two groups. Negative Responders continue to fixate the target for 1138 ms (SD 255) after naming, but Positive Responders shift gaze 332ms faster, on average at 805 ms after target onset (SD 144). The groups were comparable in their reaction times (*i.e.*, durations of first looks at distractor-initial trials), with 930 (SD 54) and with 871 (SD 95) ms, for Negative and Positive Responders respectively. Together, this shows that infants who were classified as mature segmenters at 10 months sustained their attention longer in a word-recognition task at 16 months than infants classified as immature segmenters.

**Figure 1:**
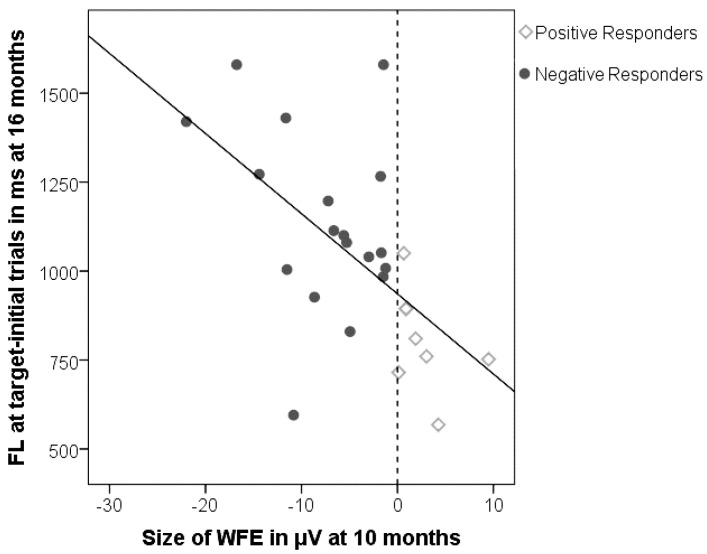
The word familiarity effect on the x-axis, and duration of first looks (FL) for target-initial trials on the y-axis: Infants with a larger negative word familiarity effect at 10 months continue to fixate targets longer at 16 months. The vertical dashed line reflects the subgroups based on polarity of word familiarity effect: Negative Responders fixate longer than Positive Responders.

This relationship is also apparent at the individual level: Figure 1 shows a strong correlation between the size of the word familiarity effect at 10 months and the duration of fixation at target after naming at 16 months (*r*_26_ = −0.58; *p* = 0.002; note that a negative correlation was predicted, given that the WFE at 10 months is on average negative in value).

Finally, we examined links with productive vocabulary sizes. Negative (mean = 25; 0–116) and Positive Responders (mean = 24; 3–44) do not differ on this measure (*t*_25_ < 1; *p* = 0.74; 95% CIs [−18; +25]). There were also no correlations between vocabulary and any of the concurrent proficiency measures from the LWL-task (*r*_26_ < 0.20; *p* > 0.32).

### 2.3. Discussion

We observed a relationship between the word familiarity effect at 10 months and a behavioral effect of word meaning recognition at 16 months: only those infants with more mature ERP correlates for word form recognition in the earlier speech segmentation task showed evidence that they accurately identified words six months later (*i.e.*, upon hearing the target label, their proportion of target looking was above chance, and they exhibited a positive difference in first looks for target-initial *vs*. distractor-initial trials). When we subsequently examined whether it was sustained attention or speed of word recognition (or both) that reflected this relationship, it became clear that it was sustained attention in which it was revealed: the larger their word recognition effect had been at 10 months, the longer infants continued to fixate the correct object upon naming.

The looking-while-listening task admits both accuracy as well as reaction time measures. Accuracy at 18–19 months has been shown to distinguish subgroups; for instance, typically-developing children from late talkers, or children who receive more child-directed speech per hour from those who receive less [44,55]. In other studies, with children at older ages, it was reaction time that was indeed related to future language profiles, and more strongly than other measures (*i.e.*, 18-month-olds: [44,55,56]; two-year-olds: [45,46]). In our 16-month-olds, accuracy as reflected by the ability to sustain attention was clearly the factor in which an earlier difference played out. Note that the ability to share attention (“joint attention”) at such an early age has been an important marker of language development [57,58]: for instance, 9–15 months old infants develop larger vocabularies when their parents are more likely to label the object their child is simultaneously fixating [59,60]. Clearly, sustaining attention is necessary for learning of early words. It may likewise be relevant for retaining and updating pre-existing memory traces. When young infants first hear words labeling novel object exemplars, increased attention will help them further consolidate the link between word and object. By a later age, infants are likely to have heard multiple tokens of these words from different speakers, and seen multiple exemplars of these objects, with the result that ease of word-to-object-mapping is established. Speed of word recognition develops more steadily once children are more experienced at mapping words to sounds [45].

Although we have shown before that markers of speech segmentation ability relate to parental ratings on vocabulary checklists [6,7,8,9], we in fact did not find such a pattern for the CDI scores in this study. Note that in this case the CDI scores were not normally distributed, and that we also did not observe any links between CDI and eye tracking performance. While many studies have used such parental reports, with positive results, there has also been some criticism, e.g., the suggestion that these ratings can be prone to parental biases [61,62]. Therefore, the lack of relationship to the CDI scores may have arisen because this measure was unreliable (although we do include this null finding in our meta-analysis in Study 3 below).

To summarize, we have demonstrated a link between early speech segmentation skill and future language development (once again) in a sample of typically-developing Dutch-acquiring infants. This finding thus fills one of the gaps in the prior literature to which we called attention in the introduction: a putative connection between brain responses to word forms in speech and later word processing skill had been hypothesized on the basis of parental reports of known words, not from actual processing evidence. The processing evidence is now in, and the predicted relationship holds, further confirming that early speech segmentation abilities scaffold later vocabulary development.

In Study 2 we turn to the other notable gap in existing research, namely that the connection of segmentation abilities to later language performance has been demonstrated for early behavioral evidence at ages from two to five, but for ERP evidence (arguably a more direct measure, *cf*. our general discussion), only up to age three so far. Study 2 will thus examine whether the link from early ERP response patterns also extends to language scores at five years.

## 3. Study 2: The Infant WFE and Language Skills at Five Years

### 3.1. Method

#### 3.1.1. Participants

Participants in the original study [10] were 28 monolingual Dutch-acquiring 10-month-olds (mean age 308 days, range 288–320 days, 11 girls). Twenty-three children (14 boys, 9 girls) were available for re-testing (a return rate of 82%, similar as in [7]). The 23 children were now on average 62.5 months old (range 55–66 months, SD = 3.7 months); none had history of seeing a speech therapist. All parents signed informed-consent forms.

#### 3.1.2. Procedure and EEG Recordings at 10 Months

Infants listened to at least nine blocks (maximum 20) of unique familiarization-and-test phases. Each familiarization phase comprised 10 tokens of the same isolated word, followed by a test phase of eight unique sentences, half containing the familiarized word. Infants were awake and seated in a child seat, facing a computer screen in a sound-attenuating booth. Each infant could watch screen savers (not synchronized with the auditory input) on a computer screen, or play with a silent toy. A parent sat by the child, listening to a masking CD through closed-ear headphones. Their EEG was continuously recorded at 200 Hz with infant-size Brain-Caps (*cf*. [7,10,28]), with 5 Ag/AgCl electrodes encompassing each quadrant of the brain (left frontal: F7, F3, FT7, FC3, C3; right frontal: F8, F4, FT8, FC3, C4; left posterior: LT, LTP, CP3, LP, P3; right posterior: RT, RTP, CP4, RP, P4; note that these are the same electrodes per quadrant as reported in [7], and as originally reported in [10]). The electrooculogram was recorded from three electrodes placed over and one under the eye to monitor blinks and eye movements. Electrodes were referenced to the left mastoid online and rereferenced to linked mastoids offline. The signal was filtered off-line at 0.1–30 Hz. Individual trials with a baseline of 200 ms were screened for artefacts from 200 ms before to 800 ms after target word onset. For each condition subject average waveforms were calculated. (For more information, see [10]: No pre-processing steps were altered from the original.)

#### 3.1.3. Procedure and Materials at 5 Years

All children undertook normed language tests (the same as used in [7]). These tests are the Dutch equivalent of the Reynell Developmental Language Scales [63]. They are suitable for children between two and six years, and norm-referenced over 1000 normally developing children. Each test distinguishes levels of difficulty, with older children starting at a more advanced level. The individual scores for each subtest are converted into language quotients (LQs), depending on the child’s age in months, with a mean of 100 and a standard deviation of 15. Children with an LQ below 85 are considered to be at risk of language impairment. The tasks were (1) “Reynell Test voor Taalbegrip” [64], measuring language reception; (2) the “sentence production” test and (3) the “word production” test, both from the “Schlichting test voor Taalproductie” [65]. Parents also completed the Dutch version of the “Speech and Language Assessment Scale” (SLAS; [66]), in which they rated their child’s development on a variety of language skills compared to ‘other children of the same age’, starting from 1 (“very poor”) to 7 (“very good”). It has five composite scales: assertiveness; responsiveness; semantics; syntax; and articulation, as well as a separate scale for talkativeness.

#### 3.1.4. Analyses

As in our earlier studies [6,7,11], we divided children into Negative and Positive Responders, based on the polarity of the individual infant word familiarity effect on left frontal electrodes (F7, FT7, F3, FC3, C3). Again, in accord with these studies, we chose the time window in which the overall group pattern was originally reported: here, 350–500 ms from target onset [10]. There were 14 Negative Responders (average word familiarity effect is −8.1 μV, SD 6.7 μV; 8 boys), and nine Positive Responders (average word familiarity effect +5.3 μV, SD 4.9 μV; 6 boys). The groups did not differ in age at 10 months (*t*_21_ < 1, *p* = 0.60), although on return Negative Responders were on average three months older than Positive Responders (*t*_21_ = 2.01, *p* = 0.06; this difference occurred because children at return were tested within a shorter time period than the original infant research; but since we used standardized tasks we could control for the age difference).

When both familiarization phase and test phase comprise continuous speech, there are clear links between infants’ WFE across phases [11], showing that a mature WFE at test is retrospectively linked to the number of tokens presented during familiarization that is required to elicit a WFE. In the original study of this follow-up [10], such an analysis was not carried out. We therefore first set out to examine whether the two responder groups differed in their familiarity response for the familiarization phase (here, words presented 10 times in isolation), by replicating the analyses of mean amplitudes for the first two (“unfamiliar”) versus the last two tokens (“familiar”) in this phase. Amplitude for the time window 200–500 ms from word onset was analyzed with repeated-measures ANOVAs, with the factors Familiarity (2), Quadrant of the brain (4) and Electrode (5; left frontal: F7, F3, FT7, FC3, C3; right frontal: F8, F4, FT8, FC3, C4; left posterior: LT, LTP, CP3, LP, P3; right posterior: RT, RTP, CP4, RP, P4) as within-subjects variables, and Responder Group as a between-subjects variable (2: Positive *vs*. Negative Responders). For all tests, we used the Huynh-Feldt epsilon correction, and we report original degrees of freedom and adjusted *p*-values. To compare how often words must be heard before a recognition response appears, we also conducted for each group post-hoc comparisons with paired *t*-tests between the first two tokens and each subsequent pair of tokens. Last, we assessed whether the groups differ on language quotients or parental ratings of language abilities at five years.

### 3.2. Results

#### 3.2.1. At 10 Months: Recognizing Words in Isolation

**Figure 2:**
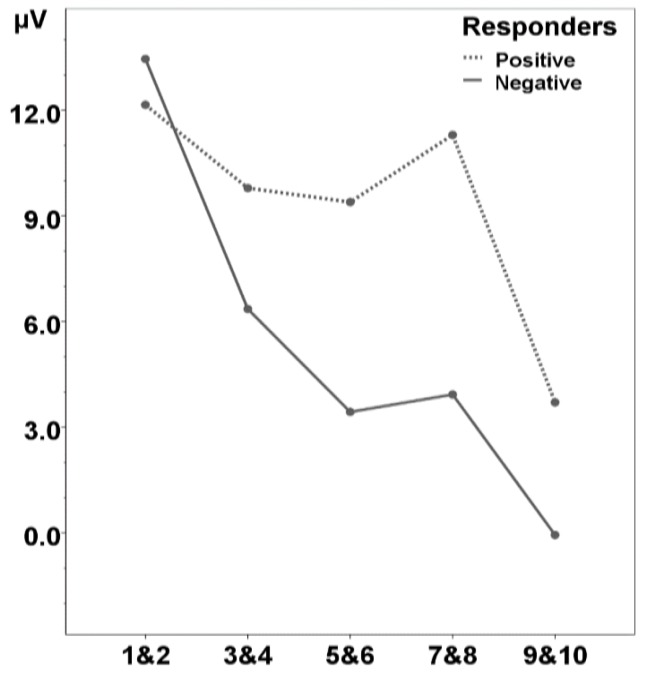
Mean amplitude in μV per word pair in the familiarization phase of isolated words from 200 to 500 ms from word onset, separate for the subgroups of infants who show a Positive or Negative word familiarity effect at test (Positive and Negative Responders, respectively).

The original study [10] examined the WFE for words heard in isolation by contrasting ERPs for the first two repetitions (unfamiliar) versus the ninth and tenth repetition (familiar). This effect was significant, over frontal electrodes, from 200 to 500 ms after word onset (N200-500). Kooijman *et al*. [10] had further observed that this effect gradually became more negative over the course of the familiarization. In the current set of analyses, we observe that Positive and Negative Responders showed similar recognition effects for the beginning *vs*. the end of the familiarization phase (*i.e.*, the interaction between Familiarity and Responder Group was not significant, F_1, 21_ = 0.23, *p* = 0.64). *Post-hoc* comparisons with paired *t*-tests, however, showed that the groups differ in the build-up of the memory trace across this phase. As Figure 2 demonstrates, for Negative Responders, the N200-500 was already significantly modulated by the third and fourth time a word was presented (*t*_13_ = 2.52, *p* = 0.026), and continued to become more negative throughout the familiarization phase (*t*_13_ > 2.20, *p* < 0.05). In contrast, Positive Responders only showed modulation when words were presented for the ninth and tenth time (*t*_8_ = 2.39, *p* = 0.044; all other comparisons were non-significant, *t*_8_ < 1, *p* > 0.4). (Note that these differences between Negative and Positive Responders were also obtained when we carried out this analysis across the full original sample of 28 infants.)

#### 3.2.2. Language Measures at Five Years

Results for the follow-up standardized language tests reveal that all children achieved scores within or above normal range. Overall, LQs were high for comprehension (m = 116.0, SD = 8.2), for sentence production (m = 117.5, SD = 8.6), and for word production (m = 117.3, SD = 9.0). Note, however, that the variation is less than in the overall population (*i.e.*, SD = 15). The SLAS average indicates that parents rated their children on average (5.2, SD = 0.7) slightly higher than their peers (corresponding to “4” on a seven-point Likert-type scale).

**Figure 3:**
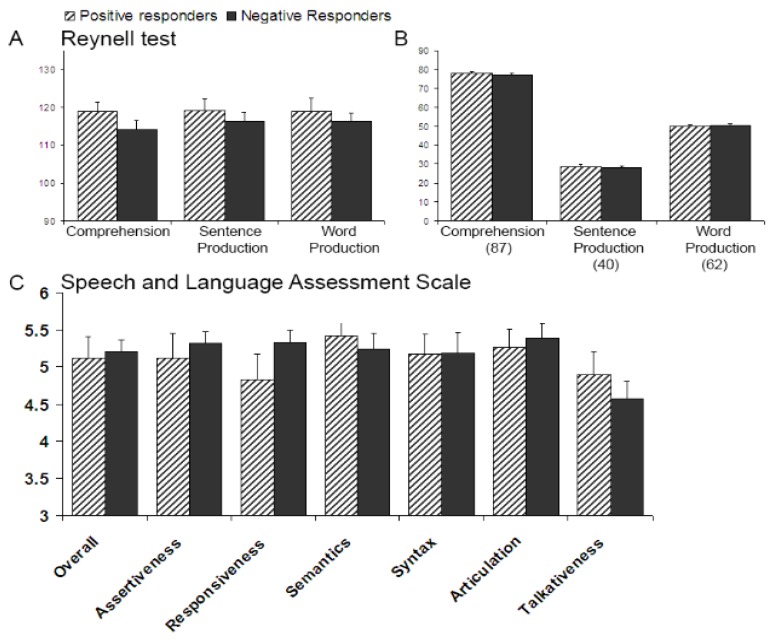
Language performances at five years split by group performance at 10 months; error bars are one standard error from the mean; (**A**) with standardized language quotients on the y-axis; (**B**) with raw language scores on the Y-axis (in parentheses the maximum possible score for each subtest), and (**C**) Mean parental ratings (overall and per subscale) on the speech and language assessment scale [66]. A score of “4” corresponds to parents rating their child’s language performance as equal to their child’s peers; higher scores reflect better language ratings.

Negative Responders have slightly lower language quotients at five years than Positive Responders (see Figure 3A; but see Figure 3B for their raw scores). These differences, however, are not significant (comprehension: *t*_21_ = 1.36, *p* = 0.19; sentence production: *t*_21_ = 0.75, *p* = 0.46, or word production: *t*_21_ = 0.70, *p* = 0.49). Hence, at five years the two subgroups have similar language profiles. Furthermore, the SLAS ratings from the parental questionnaires also reveal no Positive versus Negative Responder differences. As Figure 3C shows, parents of Negative Responders evaluated their offspring’s language abilities on average as somewhat higher than did parents of Positive Responders for their children. Nevertheless, again no differences between the groups reach statistical significance (mean rating: *t*_21_ = −0.30, *p* = 0.77); subscales (*t*_21_ < −1.4, *p* > 0.17). Correlation analyses also reveal no relationship between infant WFE and language quotients or raw scores at five years (*r*_23_ < +/−0.22; *p* > 0.31); similar analyses for the SLAS ratings of the six subscales show that the expected negative correlation is only manifested for the subscale “Responsiveness” (*i.e.*, how well the children respond to others in conversations; *r*_23_ = −0.44; *p* = 0.037; other subscales: *r*_23_ = [−28; +14]; *p* > 0.19), but note that its significance does not survive the multiple comparison correction threshold (α = 0.0083). Together, these comparisons indicate that 10-month-old Positive and Negative Responders do not significantly differ in basic language abilities at five years.

### 3.3. Discussion

This study examined whether early speech segmentation ability, as indexed in ERPs by a familiarity effect for words in continuous speech, continued to predict language development to age five. We assessed this link prospectively, by defining groups based on the polarity of the word familiarity effect at 10 months. The results do not support the existence of such a link, suggesting that early segmentation ability as reflected in the WFE may not be directly related to language profiles at five years.

However, it is clear that the presence of a WFE at 10 months is related to language profiles earlier than the age of five. In the present study, test phase WFE at 10 months was shown to correlate with the number of isolated word tokens infants needed to hear before a familiarization phase WFE appeared. Compared to the first two word tokens, infants with a mature negative WFE in the test phase showed a similar recognition effect by the third or fourth time a word was heard in familiarization. In contrast, infants with a positive-going effect in the test phase needed to hear these words nine to 10 times before showing recognition. Thus 10-month-olds with a clear WFE are at a head start compared to infants who do not show this effect. The difference between the children as measured by the standard performance scales has gone, however, by the time they are five.

In one other study [8], a relationship was indeed observed between infant segmentation ability using behavioral measures and language performance up to five years. In that study, as noted earlier, subgroups were at the extremes of vocabulary sizes at 24 months. Given that language performance varies along an extended continuum, sampling only the extremes may magnify differences which are too small to be observed at an individual level (as in our analyses) or across a larger population (e.g., had Newman *et al*. [8] included the remaining 70% of their earlier participants). Lack of variability in the standardized language tests used here cannot explain the absence of a difference in our study, given that the children made errors, *i.e.*, did not score at ceiling in these tasks (see Figure 3B). Note that we also observed no link between early word segmentation skill and later SLAS-scores. The results of the present study, then, might suggest that speech segmentation skill in infancy does not predict language skill as far ahead as five years. Other factors may well come into play by then, which could uniquely explain individual variation in language at five years, but are irrelevant for preverbal infants. For instance, attendance at (pre-) school has a strong impact on children’s development across the board (e.g., [67,68]).

Study 2 has thus provided a piece of evidence that was obviously missing from the prior pattern of results, but in doing so has raised new questions about the consistency of the observed relationships. One recommended way of examining consistency across a body of studies is to combine them in a meta-analysis. In Study 3, therefore, we undertook such a meta-analysis over all infant speech segmentation studies as a marker of language development, including the current results, with follow-up age as a moderator.

## 4. Study 3: Segmentation Skills and Later Language Skills: A Meta-Analysis

Recently, Cristia and colleagues conducted the first published meta-analysis on infant speech predictors for subsequent language development [37]. They distinguished three levels of infant markers: speech sounds, words and prosody. Vocabulary size was their outcome measure, mostly reported using the CDI [38]. All levels showed positive median weighted effect sizes of around 0.31 (95% CIs [+0.22, +0.4]), with no difference among the three linguistic levels. Their word-level studies included some on speech segmentation, but also others on word representation [69] or on word recognition with background noise [70]. To compare our results with other studies examining specifically the link between early speech segmentation ability and language development, we therefore conducted a meta-analysis across studies on speech segmentation only, adding the current results. To examine whether age at retest attenuates effect size, we further added age as a possible moderator for reported effect sizes.

### 4.1. Methods

We selected studies that report correspondences (either via correlations or subgroup comparisons) between an infant speech segmentation task and subsequent language development. These included the segmentation studies already reported in Cristia *et al.* [37] and the ones reported above; an exhaustive search in various search engines revealed no other studies that appeared between November 2012 and June 2014. (Note that three references that were included in [37] have changed in the current meta-analysis: The 2010 Junge *et al*. reference in [37] was a conference proceedings paper, with the relevant data being now in print as [7]; “Junge_2011_ch4” involved only the CDI data from Study 1, *i.e.*, not including the LWL data; “Junge_2011_ch6” now refers to Study 2 from the present report.) In total, there were six sets of unique infant samples (*i.e.*, not participating in other infant segmentation studies; with a weighted total of *n* = 192.5; note that some studies included multiple follow-up measures with different sample sizes). We obtained correlation measures either directly from result sections or calculated them from exact *t* values or raw numbers from categorical data ([28]; *cf*. [71]). Because behavioral and electrophysiological indices of speech segmentation ability have different directions of hypothesized relationships (positive and negative, respectively), our meta-analysis required that we corrected the direction of these measures: a positive correlation means that the observed relationship was in line with the hypothesis. Only one effect size per sample was considered: when multiple correlations were reported, we calculated a weighted mean r, taking into account sample size per correlation. We used the R package meta [72] to analyze data; *cf*. [71] for more details.

### 4.2. Results and Discussion

**Figure 4:**
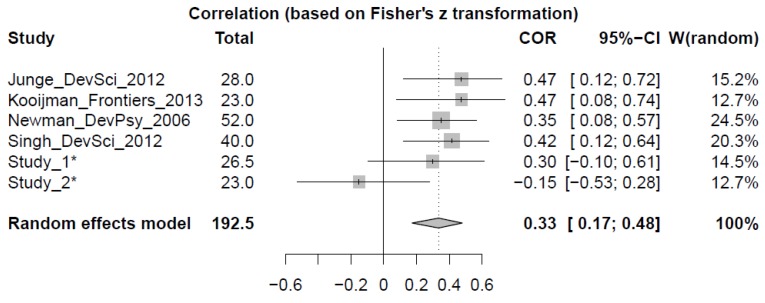
Results of the meta-analysis, with each line summarizing a study that describes links between early segmentation ability and future language scores. Studies are indicated by first author, journal and year of publication; * indicates studies presented in the current paper. “Total” gives the number of observations per study (averaged when multiple effect sizes could be calculated). The forest plot presents the weighted mean coefficient of the correlation effect sizes (“COR”; its sign corrected for hypothesized relationship), with lines spanning the 95%-confidence intervals (“95%-CI”). The scale of the forest plot is given at the bottom. “W(random)” reflects the relative weight of this study in the random effects model fit. This model is summarized in the bottom row.

**Figure 5:**
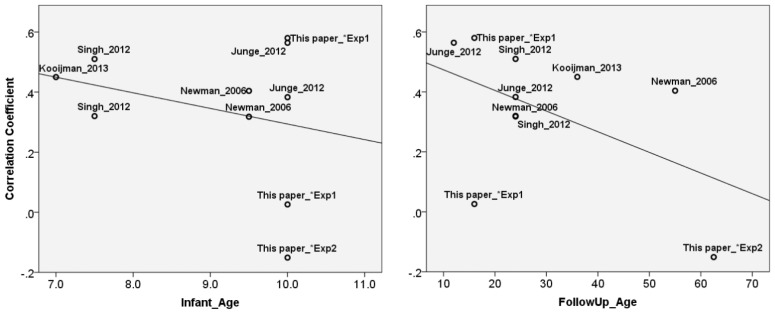
Scatterplots with correlation coefficients between infant speech segmentation ability and future language profiles plotted on Y-axis as a function of infant age (**left**), and of age at follow up (**right**). Labels next to data points refer to the studies from which they were obtained; some studies reported multiple effect sizes. Note that for event-related potentials (ERP) studies, the polarity of the correlation coefficient has been reversed to allow for comparison with the head turn preference (HTP) studies: a positive correlation indicates that the observed correlation conforms to the hypothesis.

Figure 4 plots the random effects model: the median correlation coefficient was +.3347 (95% CIs [+0.17; +0.48]), which is above zero (Z_6_ = 3.93; *p* < 0.0001). Heterogeneity statistics show that the six studies do not differ in their effect sizes (Q_5_ = 6.63; *p* = 0.25). We then examined whether age modulated effect size. Figure 5 plots each correlation coefficient reported within a sample with infant age (left), and with age at follow-up (right). Although there are relatively fewer points for later follow-up ages, the factor Age does not appear to modulate correlation coefficients in either of the two measures (*r*_10_ ≤ −0.489; * p* > 0.15).

## 5. General Discussion & Conclusions

This report has presented three studies linking individual differences in ability to recognize words from running speech in infancy to subsequent language skill at later stages of development. In the first two studies we examined this relationship prospectively, by creating subgroups based on the electrophysiological patterns associated with early word recognition; more specifically, we grouped participants based on the polarity of this signal. These two studies complement and extend previous research in a number of important ways.

Study 1 is the first investigation that reveals that electrophysiological correlates of infant speech segmentation ability predict future online language processing measures at an age in which language abilities is generally estimated by means of parental reports. Measures obtained from the “looking while listening” paradigm have been shown to be related both retrospectively and prospectively to vocabulary development from the second year onwards [44,45]. Such on-line measures are usually obtained when most children clearly understand words, *i.e.*, in the second year of life. Individual variation in this task has been interpreted partly in terms of differences in the amount of speech that children have heard from their mothers [55,73] or by differences in socio-economic status (SES; [56]). Study 1 therefore extends this earlier research, by showing that the child’s ability to understand words while speech is unfolding is also predicted by their infant skill to detect words in running speech.

Study 2 further examined the predictive validity of infant markers of speech segmentation by taking a longer view, asking whether this variability also explained language performance both in infancy and when these children were five years old. This study revealed two findings. First, the relationship was apparent in infancy: concurrent measures obtained from familiarization phases reflecting isolated word recognition skill show that those infants with mature recognition responses for words in utterances (*i.e.*, the Negative Responders) started recognizing isolated word tokens after three to four occurrences, whereas infants with less advanced recognition responses (*i.e.*, the Positive Responders) needed to hear these tokens nine to ten times before they initiated a similar recognition response. However, the second finding is that this relationship is no longer visible some four years later: the two subgroups do not differ in their language profiles at five years.

Finally, Study 3 compared the reported relationships between infant markers of speech segmentation ability with markers of future language development from the first two studies with earlier reports. A meta-analysis showed that overall, infant markers have a significant predictive value, but the analysis did not indicate that the importance of speech segmentation ability wanes over time. Not very many data points included in our analysis had been collected after three years of age, to be sure. Nonetheless, the outcome warrants a closer inspection of why Study 2 failed to show this prospective link.

The absence of a group difference at five years is in notable contrast with [7], who used exactly the same early and follow-up tasks, but measured at different ages (early task: 7 months, follow-up tasks: 3 years). It is possible that in the present Study 2 either our early task or our follow-up tasks failed to be sensitive enough to capture group differences. First consider the early task; here, 10-month-olds were familiarized with 10 instances of isolated words, then tested for familiarized words within utterances. Arguably, this task is easier for the 10-month-olds, with three months more listening experience to their native language, than it is for the 7-month-olds studied by Kooijman and colleagues. When the task becomes more difficult, for instance when familiarization is reduced to only one token, presented within an utterance to boot, individual variation in a speech segmentation task might prove more meaningful than variation in a simpler word recognition task [6]. Similarly, Singh and colleagues [9] observed that the relationship with future vocabulary sizes also hinges on the difficulty of a speech segmentation task, with the more difficult a task, the better the prediction. Moreover, in comparison to an HTP analog, this ERP task should be relatively easy: infants were only required to show a discrimination response, whereas in the HTP version, infants not only had to recognize familiar words, but also to prefer these words (*i.e.*, value them against the control words, on some scale of attractiveness), and to act accordingly (*i.e.*, to listen more, or less). Note that in such studies, both directions of preference have been reported (*i.e.*, familiarity preference *vs*. a novelty preference), although speech segmentation studies with natural speech generally report a familiarity preference (whereas studies with artificial language streams generally report a novelty preference). While it remains unclear on what grounds infants prefer some stimuli over others at a given stage of their development—due to pleasantness, familiarity and/or novelty (*cf*. e.g., [74,75,76])—it is probable that HTP is more cognitively demanding than an ERP experiment. The segmentation measure in question did however succeed in capturing a significant difference in the number of isolated tokens infants needed to hear to initiate a recognition response, as shown in the Study 2 analyses; the measure was therefore not too weak to reveal significant differences.

Consider then the follow-up tasks: a standardized language test battery (Dutch version [64,65] of the Reynell Developmental Scales [63]), applicable to children from two to six years. Our subjects, most from higher middle-class homes with college-educated parents, were just within the age limits of the task (years;months;days: range 4;7;21 to 5;6;3; mean 5;2;14). Each subtask allowed older children to start at a more advanced level, which translates as less room for variation (since there were fewer items to administer) than in the Kooijman *et al*. [7] comparison. The experimenter ended a task whenever children failed a certain number of items in a row, but this was never the case with the five-year-olds (although it happened with a majority of the three-year-olds). Hence, it could be that children performed here at ceiling. Recall however that their raw scores show non-minimal error totals (*i.e.*, around 10 per task) and that moreover the two five-year-old subgroups made a roughly equal number of errors; this suggests that there would have been room for differences to show up had they been there. The possibility remains, though, that these test tasks, especially the word-based tasks, were simply not sensitive enough to detect meaningful variation. Not all tasks explain variation in language proficiency equally well, as evidence on “late talkers” for instance shows. Late talkers are children who initially fall behind in their language production. Once at kindergarten, they tend to fall within the normal range of language skills as measured by standardized language tests (e.g., [77,78,79]. Despite this, closer inspection of their language processing often shows some residual weaknesses in their later years, in particular in verbal memory and reading comprehension [79,80,81], in comparison to children who were never delayed in word production. Standardized language tests administered to older children might thus capture gross deviations such as language impairments, but might not be sensitive enough to reflect all meaningful differences in language processing within a typically-developing population. These differences might still show up in some aspects of later language skills; indeed, even some more demanding tasks on which five-year-olds are beginning in their school classroom, such as telling a coherent story, might allow more room for subtle variation in language skills to appear.

Besides correspondences with future linguistic skill, we note that infant markers of speech segmentation ability might in principle be related with cognitive development more generally (e.g., IQ scores). There is some evidence that non-linguistic infant tasks bear on language development [82,83], though it is as yet unclear whether the reverse relationship holds. To date there have been only two studies examining this link for speech segmentation ability, with mixed results: Singh and colleagues found significant correlations for two-year-olds (for the easier speech segmentation task; [9]), whereas Newman and colleagues observed no relationship to IQ scores at five years [8]. It is certainly to be hoped that future studies of the predictive value of infant markers of speech segmentation will take into account non-linguistic as well as linguistic measures.

For a maximally sensitive test of the predictive value of early differences over time, it would certainly also be desirable to test the same infants at multiple time points across their development. Unfortunately, this was not feasible with the population we tested in the time we had. Consider again, however, the result of our meta-analysis, which compared across different studies, each with a different group of children, whether or not speech segmentation ability provided a valid marker of language processing. Reports on the prospective validity of infant markers of speech segmentation ability tend, like infant research in general, to be based on relatively small sample sizes (usually less than 50). In the long term it is very important to replicate and complement such studies. In the shorter term, a meta-analysis can be highly informative; by comparing studies on their effect sizes rather than on p-values, it goes beyond a traditional review comparing significant versus insignificant results [84]. In this case, the outcome was: attenuation over time of early differences is not supported. This result indicates that the null effect from Study 2 does not differ from all other studies that reported positive links, with vocabulary size in particular. Consistent with the conclusion that positive links with vocabulary size are to be expected are findings from other research in which differences in early vocabulary size relate to progress in grammar [85], to reading development [86,87], and to language proficiency in pre-school years [88], all of which in turn is eventually predictive of future academic careers [89,90]. On the evidence so far, infant performance in a speech segmentation task has clear predictive value for future language development.

Most parents speak to their children in multi-word utterances. Thus it is vital for every child to build on early speech segmentation abilities, and to start recognizing words in the speech stream. But nevertheless, children differ in their early segmentation performance. Finally, we consider what the source of these individual differences in speech segmentation ability might be, and whether it will prove possible to distinguish endogenous factors from environmental factors. Note that the same environmental factors (e.g., amount of input and SES) that correlate with variation in LWL tasks are also likely to correlate with endogenous factors such as variation in infant segmentation tasks. It is old news that differences in child-directed speech relate to differences in language development (e.g., [91,92,93]). However, such correlations do not entail that the environmental factors drive the causal direction. Recent research suggests, for instance, that infants themselves might actually have a considerable role in steering the clarity of their mother’s speaking style [94]. It remains unclear also at which stage(s) of language development environmental effects become apparent, and whether these effects remain stable or alter (*i.e.*, increase or diminish) over time. Environmental factors such as SES correlate with level of language proficiency at 18 months, and these effects become more pronounced at 24 months [56]. A recent review [37] suggests, though, that proficiency differences may be already noticeable in infancy (between four and 12 months), and may appear in a variety of language processing tasks (at the phoneme, word, or prosody level) that require listening experience with native language. Recall that for Dutch 11-month-olds, it was the proportion of multi-word utterances in the speech directed to them that was related to their vocabulary size at that age [5]. Thus finding words in the speech stream is crucial for vocabulary construction, which in turn is fundamental for continued language development, including grammar and reading development [85,87].

In recent years there have been a variety of speech perception tasks highlighting infant development, with some tasks showing clear correspondences with future lexical skill, such as tasks indexing speech segmentation skill, as evidenced in this paper. These relationships are observed in typically developing full-term infants, coming from families without language problems. However, recent figures estimate that over 10% of children suffer from some kind of language delay [95,96,97].

Infants at risk of delay, either because they are preterm [98], come from families with language impairments [99,100], or have siblings diagnosed with autism spectrum disorder [101,102], already show such delays from first registration point, making it hard to target interventions. To guide clinical interventions as well as provide clinically relevant new predictors at an age as young as possible, future research could therefore examine whether infant speech segmentation ability might also prove to be a reliable marker for lexical development for these infant populations at risk of language delays. Little is known about the developmental trajectories of their speech segmentation abilities. There is one study that documents pre-terms (who have an increased risk of language delay), showing that pre-terms are delayed in their ability to segment words, compared to full-term eight-month-olds matched for gestation [103]. Here, too, we see that their individual performances in speech segmentation ability predict their future lexical development: Those pre-terms with responses matching the control group have higher productive vocabularies at 12 and at 18 months. All in all, abundant demonstrations of the prospective validity of infant speech segmentation tasks have now been produced: infants who show they can recognize words from speech, and hence demonstrate that they have adequately segmented the speech stream, go on in early childhood to develop larger vocabularies at least, and potentially greater proficiency in a variety of further language skills as well.
